# Reaching Men and Women Through a Decentralized Mobile Service Delivery Model for HIV Prevention and Pre-Exposure Prophylaxis Services in South Africa

**DOI:** 10.9745/GHSP-D-24-00258

**Published:** 2026-08-10

**Authors:** Pelisa Nongena, Catherine E. Martin, Lorrein S. Muhwava, Fatima A. Cholo, Glory Chidumwa, Maserame V. Mojapele, Vusile Butler, Saiqa Mullick

**Affiliations:** a Wits RHI, University of the Witwatersrand, Johannesburg, South Africa.

## Abstract

**Introduction:**

Structural and service delivery barriers such as long waiting times, health care provider attitudes, and distance from services prevent access to HIV prevention and pre-exposure prophylaxis (PrEP) services, particularly for young people. The World Health Organization recommends differentiated service delivery models for HIV prevention and treatment services, including through mobile outreach. Mobile health clinics offer an opportunity to overcome barriers to service access by bringing services closer to places of work or study, improving convenience, minimizing stigma, and expanding the choice of service delivery locations and types.

**Methods:**

We conducted a mixed-methods study describing a mobile service delivery model for HIV prevention and PrEP services in 3 provinces in South Africa. The study used routine program monitoring and evaluation data from 35,375 men and women aged ≥15 years accessing services at project fixed facility and mobile clinic sites between January 2019 and October 2022, in addition to data from 74 in-depth interviews with a purposively selected sample of adolescent girls and young women aged 15–24 years and men aged ≥15 years enrolled in a nested study cohort.

**Results:**

Overall, 11,687 people were reached through mobile clinics between January 2019 and October 2022, of whom the majority were female (89.3%) and between 15–24 years (82.8%). Nearly all (96.0%) mobile clinic clients received an HIV test and 80.8% initiated oral PrEP, similar to HIV testing and PrEP initiation rates at the fixed facilities. Health care users demonstrated a high level of satisfaction with mobile health services, with benefits including a reduction in trips to fixed facilities, improved privacy, and reduced waiting times. However, some participants noted challenges with mobile services, including inconsistent schedules.

**Conclusion:**

Mobile health services with youth-friendly staff have the potential to reach young people in need of HIV prevention services and are feasible and acceptable in the South African setting. Mobile health services offered benefits such as convenience, privacy, and reduced need to travel to clinics, although they could be enhanced through improved consistency of services.

## Abstract (precis)

Decentralized mobile health services were successful in reaching young people with HIV prevention and pre-exposure prophylaxis services in South Africa, offering benefits such as convenience, privacy, and reduced need to travel to clinics.

## Teaser

**Key Findings:**

- Decentralized mobile health services, delivered in a youth-friendly manner, were successful in reaching and providing young women with HIV prevention and oral pre-exposure prophylaxis (PrEP) services.
- The benefits of mobile health services included convenience, privacy, reduced waiting times, and reduced need to travel to clinics, although they could be enhanced through improved consistency.

**Key Implications:**

- Mobile health services provide an additional service delivery channel to reach young women with HIV prevention and PrEP services, in a way that responds to their needs and preferences.
- Decentralized service models may expand opportunities to improve access to and uptake of HIV prevention services, as well as to other sexual and reproductive health services.
- Having a choice of service delivery options will remain an important component of service acceptability.

## INTRODUCTION

South Africa has an estimated HIV prevalence of 12.7%, with approximately 7.8 million people living with HIV.^1^ HIV prevalence is twice as high among adolescent girls and young women aged 15–24 years than among males of the same age, and threefold higher among women 25–29 years compared to their male counterparts.^1^ National guidelines support the provision of a package of HIV prevention services, including the use of pre-exposure prophylaxis (PrEP), condoms, and voluntary medical male circumcision (VMMC), as well as access to HIV testing and linkage to antiretroviral therapy (ART).^2^

However, literature indicates a gap in young people’s health-seeking behaviors,^3^^,^^4^ and there remain multiple barriers to accessing HIV prevention and PrEP services. Notwithstanding product-related barriers to PrEP uptake and use,^5^^,^^6^ studies have reported a number of structural and service delivery-related barriers. These include long waiting times, attitudes of health care providers, lack of privacy, inconsistent availability of medicines, inconvenient operating hours, and distance from services.^7^^,^^8^ Addressing access challenges is a key consideration for PrEP uptake and use among young people.^7^

The World Health Organization (WHO) recommends differentiated service delivery models for HIV prevention and treatment services, including through private or community pharmacies, mobile outreach, and home delivery.^9^ Client-centered differentiated models of care may increase acceptability and accessibility of PrEP while also alleviating the burden on the health system.^3^ Mobile health clinics offer an opportunity to overcome barriers to sexual and reproductive health (SRH) and PrEP service access, by bringing services closer to places of work or study, improving convenience, minimizing stigma, and expanding the choice of service delivery locations and types.^9^

In prior research, mobile health services have shown acceptability and success in delivering and improving access to contraceptives, treatment for sexually transmitted infections (STIs), and related services for young women.^10^^–^^13^ In addition, studies have reported on the acceptability^12^^,^^14^ and cost-effectiveness^15^ of mobile clinics for HIV testing services in facilitating testing of younger people^12^^,^^14^^,^^16^ and people living with HIV (PLHIV) earlier in their disease course in South Africa.^17^ A study describing PrEP delivery through mobile clinics in Cape Town, South Africa, found the model to be acceptable and accessible to adolescent girls and young women.^18^ In addition, a study conducted in the Eastern Cape in South Africa found higher rates of PrEP initiation when services were co-located with community testing compared with referral, indicating the acceptability and possible preference for community-based models.^19^^,^^20^

Nevertheless, there remains limited evidence on the provision of mobile service delivery models for HIV prevention and PrEP services at scale. This article adds to existing knowledge by describing a mobile clinic model for the delivery of HIV prevention services for adolescent girls and young women, including daily oral PrEP, designed and implemented as part of the Unitaid-funded Wits RHI Project PrEP in South Africa.

## MATERIALS AND METHODS

### Study Setting

Project PrEP is an implementation science study, which has generated knowledge to inform the introduction and integration of oral PrEP as part of combination HIV prevention and SRH services for adolescent girls and young women in South Africa.^21^ The study began in 2018, at the time that oral PrEP was beginning to be introduced nationally in public health care facilities. The project is implemented in 4 geographical areas that were selected in collaboration with the Department of Health (DOH) based on the burden of HIV and population of young people in the surrounding communities. Each project area consists of 2 DOH primary care fixed facilities and 1 roving mobile clinic. They comprise of 1 peri-urban area in a residential township outside of Gqeberha in the Eastern Cape, 1 rural area in the town of Mthatha in the OR Tambo district in the Eastern Cape, 1 urban area in eThekwini in Kwa-Zulu Natal, and 1 peri-urban area in a residential township of Tshwane in Gauteng. Antenatal HIV prevalence rates in these areas in 2019 ranged from 23.1% in Tshwane, 29.7% in Gqeberha, 35.2% in Mthatha, and 43.5% in eThekwini.^22^

HIV prevention and oral PrEP services are provided in line with national guidelines and through a combination package of HIV testing, contraception, STI management, and provision of condoms, with linkage to other prevention or treatment services as required.^2^ HIV testing is conducted by trained lay counselors, and oral PrEP is prescribed by a nurse trained in Nurse Initiated Management of Antiretroviral Therapy (NIMART), in line with local policy. All services are provided free of charge to clients. Demand generation for HIV prevention services is facilitated through collaborations with local community-based organizations and through the use of national online digital platforms that benefit young people (bwisehealth.com and myprep.co.za).^23^^,^^24^

### Development and Description of the Mobile Service Delivery Model

**FIGURE 1:**
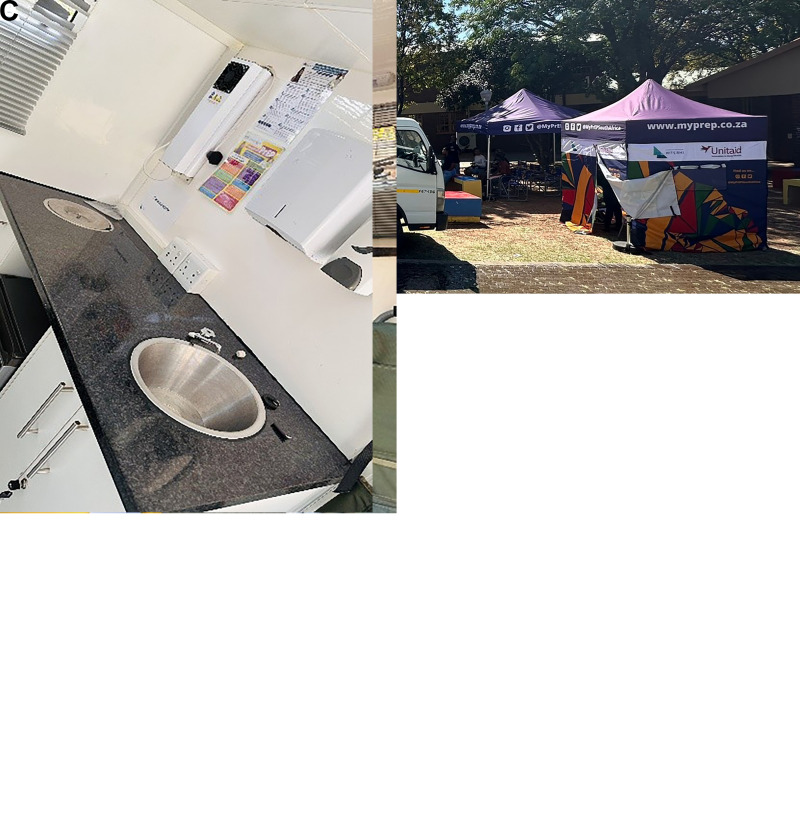
Project PrEP Mobile Clinic Van, South Africa, 2024

Given the potential that mobile service delivery models had shown to overcome barriers to SRH service access in a way that was acceptable to young people, the project embarked on a process to expand the traditional, fixed clinic service delivery model in project sites to include a roving mobile clinic. The mobile clinic was also intended to facilitate ease of access to services in locations where young people were located, such as tertiary education institutions, while offering clients a choice of access to services through fixed or mobile clinic service points. In collaboration with the DOH and with input from young people in the surrounding communities, Project PrEP developed a service delivery model that would be acceptable and accessible and that would overcome some of the structural barriers to HIV prevention service access for young people. The inclusion of a mobile clinic service delivery component aimed to supplement and extend the reach of the fixed facilities and to improve convenience and access to SRH services, with a focus on reaching adolescent girls and young women. One mobile clinic was allocated to each of the 4 project areas, roving within the catchment areas of the fixed facilities to which they were linked. Mobile services began operations in Gqeberha in January 2019, Tshwane in June 2019, eThekwini in August 2019, and Mthatha in December 2019. Branding of the clinic was developed in collaboration with the DOH and aligned to the branding of the She Conquers campaign, a national campaign aimed at reducing HIV infections, pregnancy, and gender-based violence (GBV) among adolescent girls and young women, and retaining girls in school.^25^ The branding aimed to be youth-friendly and not to focus specifically on SRH or HIV, thus avoiding stigma associated with HIV services (Figure 1). In addition, the project was guided by a national adolescent and youth-friendly service approach and training was provided to all staff members, ensuring that services were accessible and responsive to young people. The project also specifically employed young people as peer navigators and demand creation officers to facilitate and support access to services for young people.

The mobile clinics were adapted from 6-ton trucks, each with 3 consulting rooms (Figure 1). To facilitate comprehensive SRH clinical service provision and to ensure compliance with requirements regarding storage of medicines, mobile clinics were each set up with a small refrigerator, air conditioner, mounted drug cupboard, examination bench and set, sink with water supply, and toilet. The mobile clinic accommodates 2 clients and 2 staff members at a time, while ensuring privacy and confidentiality for the client. In line with local requirements for HIV testing and PrEP services, a lay counselor provides HIV counseling and testing in the first consulting room and a nurse provides clinical services in the second room, with the third room used to collect specimens if required. Clinical services include on-site provision and dispensing of oral PrEP, contraception (oral and injectable methods and condoms), and STI syndromic screening and treatment, with commodities supplied by the fixed facility to which each mobile is linked. Additional staff are stationed in gazebos set up alongside the mobile clinic, and include a peer navigator who provides health talks and navigates clients to access the service provision point, and a data capturer.

**FIGURE 2:**
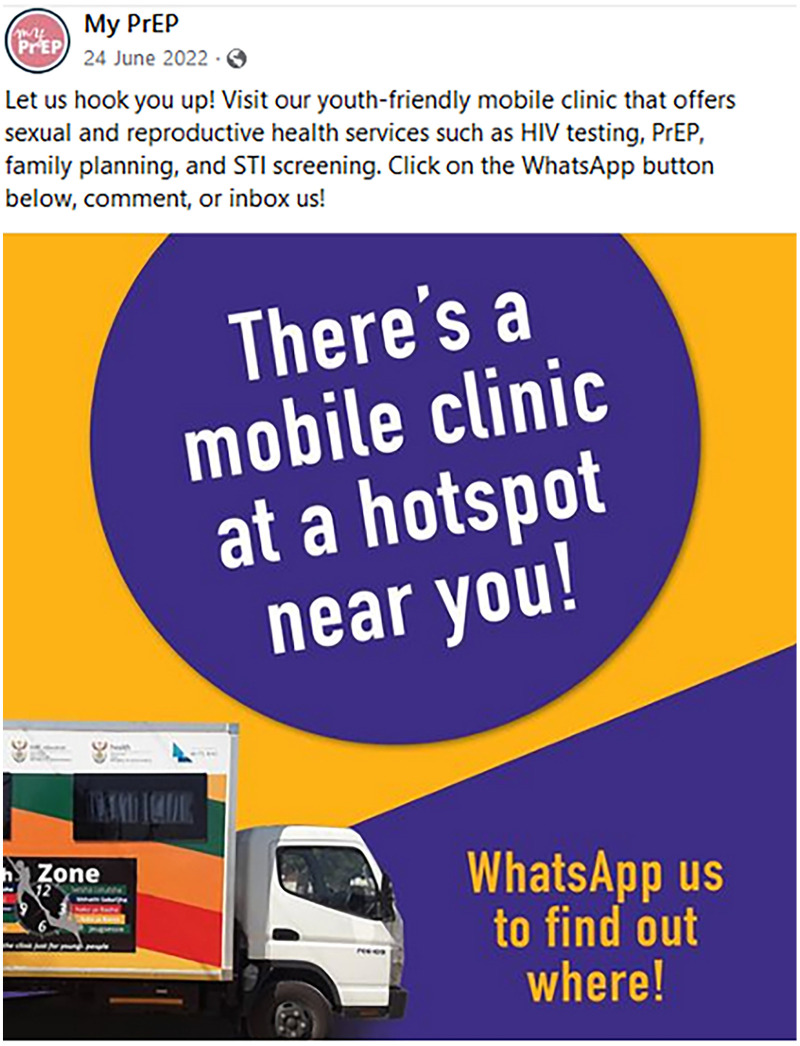
MyPrEP Facebook Post Announcing the Mobile Clinic Schedule, South Africa, 2022

Service delivery locations for the mobile clinic were together identified by the project team, staff at the health facility and young people, selecting places within the clinic catchment area where young people are known to work, study or socialize, such as institutions of learning, bus stops, taxi ranks, shopping malls, informal trading areas, and open spaces in the community. Services were provided at varying locations on weekdays and weekends, according to a schedule that ensured each location was visited at least once or twice a month. A day before the mobile was scheduled to provide services, staff visited the community to create awareness that the mobile clinic would be in the area providing services. In addition, the mobile clinic schedule was shared on social medial platforms, such as the MyPrEP Facebook page (Figure 2), and through temporary signage in the vicinity of the service point.

### Challenges to Mobile Service Delivery

There were several logistical considerations and challenges to providing mobile clinic services, which were identified during service implementation. These included the availability of suitable space to park and locate the mobile clinic and linked gazebos. This was more limited in highly urbanized areas, but easier to accommodate where space for services could be negotiated with a partnering organization, such as learning institutions or shopping malls with sufficient open spaces. Adherence to bylaws and requirements for permits to park the mobile clinic also needed to be navigated and at times posed a limitation to service delivery. In addition, safety challenges were observed, particularly in high-crime areas, at times compromising the ability of the project to provide services as scheduled or requiring service provision to be concluded early to ensure staff were able to complete their work during daylight hours. Furthermore, theft of essential equipment and vandalism resulted in halted services and increased costs, alongside the need for increased maintenance and replacement of parts as the mobile clinics aged. Extreme weather conditions such as heavy rain or intense heat also hindered operations, as the awnings used to provide shade for clients waiting for services proved inadequate, leading to clients leaving without receiving services or not attending at all.

### Data Collection and Analysis

#### Study Design and Population

This was a mixed-methods study that used routine program monitoring and evaluation data from men and women accessing services at Project PrEP fixed facility and mobile clinic sites, along with in-depth interviews (IDIs) with a purposively selected sample of adolescent girls and young women and men who had been enrolled in a nested study cohort. Data were collected between January 2019 and October 2022. Routine program data were collected from all clients ≥15 years old accessing services at Project PrEP fixed or mobile clinic sites. The nested cohort comprised HIV-negative females aged 15–24 years and HIV-negative males aged ≥15 years, initiated on oral PrEP at a project site. A subset of these participants were eligible for participation in an IDI if they were female aged 15–24 years or male aged ≥15 years, HIV negative (as determined by a point-of-care rapid HIV test), initiated oral PrEP at one of the project sites, and able and willing to provide written informed consent.

#### Program Data Collection and Analysis

Routine program data included client demographics and socio-behavioral characteristics recorded in clinic files by the consulting nurse. These included gender, age, employment status, relationship status, partner’s age, main partner’s HIV status, STI symptoms, transactional sex (defined as having sex in exchange for money/gifts/clothes in the preceding month), sex under the influence of drugs and alcohol, consistent condom use (defined as using a condom every time they had sex in the preceding month), and whether they had ever tested for HIV. Data on provision of rapid point-of-care HIV testing and initiation of oral PrEP (defined as a documented script for oral PrEP) were also recorded.

We conducted a descriptive analysis of clients who accessed services at Project PrEP sites, by service delivery type. Data were collected from 35,538 clients who accessed services from Project PrEP sites between January 2019 and October 2022. Of these, data for clients with missing age data (n=163) were excluded, leaving a final sample of 35,375 participants. Data were analyzed using Stata statistical software, version 15.0.^26^

#### Qualitative Data Collection and Analysis

Data from IDIs with 44 female (15–24 years) and 30 male (≥15 years) PrEP users purposively sampled from the nested cohort were analyzed. A combination of face-to-face and telephone interviews were conducted by trained researchers and fieldworkers between February 2020 and May 2022, approximately 1 year after the initiation of project activities. PrEP users included those newly initiated on PrEP, those who were currently using PrEP, and those who had interrupted or discontinued PrEP use, to gain a diversity of views. Interviewer-participant pairs were matched by gender and participant’s preferred language. A semi-structured interview guide was used to guide the discussion and explore participants’ experiences of PrEP use and the barriers and facilitators to PrEP access. The IDIs were audio recorded and ranged between 15-120 minutes. Audio recordings were transcribed, translated, and reviewed for accuracy prior to data analysis. For this article, we conducted an inductive analysis of the qualitative data using NVivo 12 software to draw out participants’ experiences of accessing PrEP services as well as the preferred service delivery models (i.e., fixed facility and mobile clinics).

### Ethical Considerations

This study was approved by the Human Research Ethics Committee at the University of the Witwatersrand (M180860) and the World Health Organization (WHO) Ethics Research Committee (Wits-PrEP-AGYW). Study participants provided written informed consent for participation in the IDIs. Minors provided independent consent if they demonstrated understanding of the study parameters; alternatively, parental consent and assent were provided.

## RESULTS

There were 35,375 clients who received services at Project PrEP sites between January 2019 and October 2022. The demographic and socio-behavioral characteristics of all clients are presented in the Table. Of all clients, 33.0% (11,687/35,375) accessed services through a mobile facility. Among those accessing mobile services, the majority (89.3%) were female, and most (82.8%) were 15–24 years old. The percentage of clients accessing services through a mobile clinic ranged from 7.2% in eThekwini to 25.8% in Gqeberha, 29.6% in Tshwane, and 37.4% in Mthatha. The majority of clients seen in mobile clinics were currently studying (82.3%) and in a relationship (75.3%). Of those with a partner, the majority did not know their main partner’s HIV status, higher among mobile clinic clients (62.0%) than those at fixed facilities (49.9%). Of mobile clinic clients, 9.6% reported STI symptoms, 2.7% had engaged in transactional sex, and 5.2% had sex under the influence of alcohol or drugs. Just 20.6% reported consistent condom use. Most clients seen at the mobile clinic had tested for HIV before (83.5%), although the proportion was lower than among those accessing fixed facilities (88.0%). Almost all clients seen at the mobile clinic (96.0%) received an HIV test and 80.8% initiated oral PrEP, similar to the proportion accessing HIV testing and PrEP initiation at the fixed facilities.

**TABLE.** Demographic and Socio-Behavioral Characteristics of Clients Accessing Services at Project PrEP Sites by Type of Facility, South Africa, January 2019–October 2022

	**Fixed Facility** **(N=23,688)**		**Mobile Facility** **(N=11,687)**		**Total** **(N=35,375)**	
	**No.**	**%**	**No.**	**%**	**No.**	**%**
**Gender**						
Male	3,742	15.8	1,245	10.7	4,987	14.1
Female	19,932	84.1	10,435	89.3	30,367	85.8
Transgender	14	0.1	7	0.1	21	0.1
**Age group**						
15–17 years	3,779	16.0	2,433	20.8	6,212	17.6
18–20 years	5,979	25.2	3,196	27.3	9,175	25.9
21–24 years	8,229	34.7	4,050	34.7	12,279	34.7
25–34 years	4,049	17.1	1,710	14.6	5,759	16.3
≥35 years	1,652	7.0	298	2.5	1,950	5.5
**Geographical area**						
Gqeberha	4,408	18.6	3,020	25.8	7,428	21.0
Tshwane	5,113	21.6	3,460	29.6	8,573	24.2
eThekwini	7,881	33.3	838	7.2	8,719	24.6
Mthatha	6,286	26.5	4,369	37.4	10,655	30.1
**Employment status**						
Employed	4,884	20.6	861	7.4	5,745	16.2
Unemployed/not in school	7,362	31.1	1,209	10.3	8,571	24.2
Scholar or studying	11,442	48.3	9,617	82.3	21,059	59.5
**Relationship status**						
Single	3,509	14.8	2,576	22.0	6,085	17.2
In a relationship	19,198	81.0	8,805	75.3	28,003	79.2
Open/casual relationship	864	3.6	280	2.4	1,144	3.2
Unknown	117	0.6	26	0.3	143	0.4
**Partner’s age difference** ^a^						
≤5 years	15,341	76.5	7,374	81.2	22,715	77.9
>5 years	3,687	18.4	1,336	14.7	5,023	17.2
Unknown	1,034	5.2	375	4.1	1,409	4.8
**Know main partner’s HIV status** ^a^						
Yes	10,063	50.1	3,453	38.0	13,516	46.3
No	9,999	49.9	5,632	62.0	15,631	53.7
**STI symptoms reported**						
Yes	1,741	7.3	1,119	9.6	2,860	8.1
No	21,947	92.7	10,568	90.4	32,515	91.9
**Transactional sex**						
Yes	1,134	4.8	295	2.7	1,429	4.2
No	20,069	84.7	11,125	95.1	31,194	88.1
Unknown	2,485	10.4	267	2.2	2,752	7.7
**Sex under the influence of drugs or alcohol**						
Yes	1,541	6.6	603	5.2	2,144	6.1
No	19,667	83.0	10,820	92.5	30,487	86.1
Unknown	2,480	10.4	264	2.3	2,744	7.8
**Consistent condom use**						
Yes	4,775	20.1	2,410	20.6	7,185	20.9
No	3,596	15.1	1,326	11.3	4,922	14.3
Sometimes	12,451	52.1	6,373	54.5	18,824	54.6
Unknown	2,866	12.0	1,578	13.5	4,444	10.2
**Ever tested for HIV**						
Yes	20,865	88.0	9,760	83.5	30,625	86.5
No	2,359	9.9	1,634	13.9	3,993	11.2
Unknown	464	1.9	293	1.3	757	0.02
**Tested for HIV at this visit**						
Yes	23,100	97.5	11,223	96.0	34,323	97.0
No	588	2.5	464	3.9	1,052	3.0
**Initiated oral PrEP**						
Yes	19,462	82.2	9,441	80.8	28,903	81.7
No	4,226	17.8	2,246	19.2	6,472	18.3

^a^ Among those with a partner (n=29,147).

Abbreviations: PrEP, pre-exposure prophylaxis; STI, sexually transmitted infection.

Participants had differing preferences for fixed and mobile service delivery models. Themes relating to a preference for mobile services included their convenience and accessibility, privacy and confidentiality, and the visual appeal of the mobile trucks. In contrast, inconsistent and unpredictable scheduling and lack of communication were themes identified as barriers to mobile clinic services. Preferences for fixed facility services related primarily to their reliability, whereas stigma, a lack of privacy, and long waiting times were identified as disadvantages of fixed facilities. With both models, positive attitudes of health care providers were identified as an important component of the service model.

### Convenience and Accessibility of Mobile Services

Some participants preferred mobile clinics within their local communities or near their schools as they offered convenience and discretion, and they eliminated the need for frequent clinic visits. They felt the mobile clinic was often less busy, and they could be assisted within a short amount of time without waiting in long queues or needing to travel long distances or pay for transport to a fixed facility.

*I would like to get service from mobile taking into consideration the convenience. If they can come more often because most of the students are attending, also some schools are far from each other. (Female, 20 years old)*

*I would like it if the PrEP truck [mobile clinic] went next to Shukushukuma [name of place] and give out the pills and leave. Instead of having to come to the facility for PrEP every month. (Female, 18 years old)*

*I think it’s better at the truck [mobile clinic]. Yes, I like it because I don’t like long queues, you see? (Male, 30 years old)*

*Firstly, we must use the mobile cars [mobile clinics], you see? They should be close to schools and be close to [their] homes. You see? It’s like they like motivating each other and go there, closer, because you find that the mobile is not busy. Yeah, they know how to motivate each other to go there, check [test] and come back. Then they will know their statuses, then access services. (Male, 40 years old)*

However, it was noted that while mobile clinics that provided PrEP services near school premises were ideal for young people, safety concerns for mobile clinic staff working in communities where protests were prevalent were justified.

*They used to come at school, that is also good. The last time I checked they were afraid of the strikes but now things are much better, I think they will come. And they always come with the clinic. (Female, 24 years old)*

### Privacy and Confidentiality of Mobile Services

Mobile clinics were also preferrable for clients who were concerned about stigma and privacy, as they were seen to be more discreet than fixed facilities.

*I think a mobile clinic near a school gate will do … I remember there was one of my friends who was hiding from her neighbor for the fear of being seen in the clinic. She knows that when she is seen by the neighbor, the neighbor will tell her mom and they would start enquiring what she had gone to the clinic to do … clinic does not work for us because apparently clinic is bad and stigmatizing. (Female, 19 years old)*

### Visual Appeal of the Mobile Trucks

The visual appearance of the mobile truck together with positive provider attitudes also influenced participants’ experience of mobile service delivery.

*PrEP staff are very friendly, you see? So, they were approaching us and the bright colors of the truck [mobile clinic] also attracted me and I was interested to know what they were doing there. (Male, 35 years old)*

### Inconsistent and Unpredictable Mobile Schedules

Inconsistent and unpredictable schedules were identified as challenges and barriers to mobile service access. A participant who had anticipated accessing PrEP services from the mobile clinic highlighted this challenge, particularly during school holidays, and how this had contributed to his discontinuation of PrEP.

*You had mobile clinics here, you see? So, I thought it would be easier to have a mobile clinic than going to the clinic straight, you see? So, by then I started taking PrEP that was late last year and then the schools were closed … the mobile clinic was supposed to come back but it didn’t come back and by then we were busy with exams. I just went home and then that’s when I stopped taking PrEP. (Male, 23 years old)*

### Need for Clear Communication About Mobile Clinic Scheduling and Location

Participants also highlighted a need for consistency and improved communication regarding mobile clinic visits and their exact location within the community to enable young people to easily locate the mobile clinic and access services.

*I think maybe that the truck comes to this location every Friday or maybe two times a month that will make it easy, and then also when the truck arrives they show us where specifically they are because we from different locations, you don’t know this kasi [colloquial term for township] or what is happening, where the clinic is, where the mall is, you see? So, those are the difficulties, actually navigating where the truck is, the consistency. (Male, 23 years old)*

### Reliability of Fixed Facilities

On the other hand, some participants preferred to access PrEP from fixed clinics, which were perceived as a more reliable service delivery point than mobile clinics, where there was a possibility of missing the mobile clinic and therefore not accessing necessary services.

*I prefer getting it [PrEP] from the clinic because the mobile clinic, I think if it was said that there are a lot of mobile clinics in all areas. But for now, I am okay with fetching it straight from the clinic because you know exactly where to go, you do not have to wait for the mobile clinic to come on whatever day it comes on, and I never see it around. So, at the clinic PrEP is always available. (Female, 20 years old)*

### Stigma Experienced at Fixed Facilities

A noted disadvantage of fixed facilities was the potential for stigma and a lack of discretion in that there was always the possibility of being recognized by community members who may make assumptions about personal reasons for clinic visits.

*But at the clinic it’s like, when you come it is known that you are sick. Or maybe you came to collect pills for the disease. Just like now, when I got here, there are people who know me - Hai, what do you want? Things like that, you see? I say, hai! No, I am going upstairs, there is a doctor that I am going to see. Because they see that I pass the queue, but if I was queueing with them, they were going to gossip, “Hai, he is sick,” and things like that. It is not like we are scared, if we are sick, we are sick, but there should not be anyone talking about it to other people, yeah. (Male, 40 years old)*

### Long Waiting Times at Fixed Facilities

One of the commonly cited challenges with fixed facilities was the long waiting time at the pharmacy. Although young people were prioritized to see the nurse, they still had to wait in long queues along with the general clinic population to receive their PrEP.

*The only problem that I had was that the medicine is supplied at the side where everyone is queuing. You see? I think that becomes a challenge because for instance, I work with time so, I travel far, like for instance, now my date is on 7th, I am in a different location so I must then come again and collect it and queue with people at the clinic who came to collect other things. That is the only thing that inconveniences me, such things. So, I think it would be right if you collect it here, and do your things here you see, and get it here, PrEP, you see? (Male, 35 years old)*

### Positive Health Care Provider Attitudes

For some participants who had accessed both types of facilities, they experienced no difference between the fixed facility and mobile clinic. These participants highlighted the importance of positive provider attitudes and that the communication they received from the providers were the same regardless of the service delivery model used.

*Here there is no difference because when I take my pills here [mobile clinic] and I take them there [fixed clinic], like the communication. The nurse is able to communicate and advise me that I should also use a condom. Don’t use this thing [PrEP] only. Please use a condom. Even when I come here [mobile clinic] they are also saying the same thing. (Male, 21 years old)*

*… they referred me to [name of fixed facility omitted]; it became so nice there because the people working with PrEP are very nice, I think all of them even those who work at the truck [mobile van] they are very nice. The people working with PrEP are very nice. (Male, 35 years old)*

## DISCUSSION

This large implementation science study, from 3 provinces in South Africa, highlights the role that mobile health services may play in reaching people, particularly adolescent girls and young women, with HIV prevention and PrEP services. In this study, more than 11,000 individuals were reached through mobile health services, of whom the majority were adolescent girls and young women, and 80% initiated oral PrEP. We found that those reached through mobile services were predominantly students and needed HIV prevention services, having high rates of inconsistent condom use and almost half not knowing their partner’s HIV status. Health care users demonstrated a high level of satisfaction with mobile health services, with benefits noted to include reduced waiting times, elimination of expensive trips to fixed facilities, and improved privacy. However, some participants noted challenges with mobile services, including inconsistent scheduling leading to poor reliability.

The findings in this study support existing findings, which have reported the role that mobile health clinics and community-based services may have in reaching young people with HIV prevention services.^12^^,^^27^ Mobile health clinics supplement fixed-facility services while also providing a choice of service delivery options for young people. These findings also substantiate the feasibility and acceptability of mobile health service delivery models for young people in the South African context. Compared to 10% among clients accessing services at fixed facilities, 14% of those accessing services at the mobile clinic had never tested for HIV before. This highlights the potential of mobile clinic services in reaching people who are less likely to engage with health care services and in offering an entry point into health care services for underserved populations.

Barriers to PrEP service access documented in existing literature, such as lack of confidentiality, long waiting times,^8^ scheduling conflicts, and time required to attend the clinic,^28^^,^^29^ may be overcome by mobile health services, which were perceived by our study participants to have reduced waiting times and improved privacy and convenience. The high uptake of HIV testing and oral PrEP among clients accessing mobile services in this study, within an integrated SRH service package, was encouraging and speaks to the acceptability and potential that mobile clinics may have in improving access to PrEP. However, we also found that there may be those who prefer a traditional clinic-based service delivery model, illustrating that having a choice of service delivery options will remain an important component of ensuring access to and acceptability of HIV prevention services.^9^ Regardless of service delivery model, the need for services to be provided in a manner that is respectful and youth-friendly was apparent and has also been highlighted in other studies as a facilitator of PrEP access.^8^^,^^30^

Although mobile clinics were acceptable to health care users, it is critical that their implementation includes clear scheduling, creation of awareness of the service, and regular communication of mobile clinic location and operating hours, as well as an assurance of service consistency. The variations in the number and proportion of clients accessing mobile clinic services between geographical areas highlight the role that contextual factors are likely to play in delivery of mobile services. For example, the lower proportion of clients accessing services through the mobile clinic in the more urbanized eThekwini project area may have been a result of more limited space and fewer feasible locations within which to provide mobile services at the time. An in-depth, local understanding of these factors will be important in determining the most suitable models of delivery for different communities.

The predominance of females reached through the mobile services likely reflects the overall focus of the project on adolescent girls and young women, with demand generation activities primarily focused on providing information and driving demand for services among this population. Previous studies have highlighted the role that mobile clinics may play in reaching men,^17^ and further evaluation is needed to determine if similarly focused models could reach more men with HIV prevention services.

### Limitations

This study has some limitations. We did not assess continued, effective use of PrEP after initiation, a challenge that has been noted in several studies^31^^,^^32^ and which requires further research into the role that mobile clinic services may play in ongoing PrEP access and use. In addition, the study did not explore health care provider perceptions of mobile clinic service delivery or analyze the cost-effectiveness of the model. These are areas that should be explored further in future research.

## CONCLUSIONS

Differentiated and simplified models of PrEP delivery have been recommended to improve the accessibility and acceptability of PrEP services, as well as to support uptake and effective use.^9^ This implementation study found that mobile health services with youth-friendly staff have the potential to reach young people in need of HIV prevention services, and they were feasible and acceptable in the South African setting. Mobile health services offered benefits such as convenience, privacy, and reduced need to travel to clinics, although they could be enhanced through improved consistency of services.

## Notes

**Peer Reviewed**

**First Published Online:** July 21, 2026.
